# Supplementary material for: Live Cell Poration by Au Nanostars to Probe Intracellular Molecular Composition with SERS
Source: Nanomaterials (Basel). 2021 Sep 30;11(10):2588. doi: 10.3390/nano11102588 (PMC8539561; doi:10.3390/nano11102588)
Supplement: Supplementary file 1 [file nanomaterials-11-02588-s001.zip › nanomaterials-1351616-supplementary.pdf]

nanomaterials

MDPI

Supplementary Materials

Live Cell Poration by Au Nanostars to Probe Intracellular Molecular Composition with SERS

Evelina I. Nikelshparg, Ekaterina S. Prikhozhdenko, Roman A. Verkhovskii, Vsevolod S. Atkin, Vitaly A. Khanadeev, Boris N. Khlebtsov and Daniil N. Bratashov

[1] Department of Biophysics, Biological Faculty, Moscow State University, 1–12 Leninskie Gory, 119991 Moscow, Russia
[2] Science Medical Center, Saratov State University, Astrakhanskaya, 410012 Saratov, Russia; prikhozhdenkoes@sgu.ru (E.S.P.); r.a.verhovskiy@sgu.ru (R.A.V.); atkin.vsevolod@gmail.com (V.S.A.); khlebtsov_b@ibppm.ru (B.N.K.)
[3] Institute of Biochemistry and Physiology of Plants and Microorganisms, Russian Academy of Sciences, Prospekt Entuziastov, 410049 Saratov, Russia; khanadeev_v@ibppm.ru
[4] Veterinary Medicine and Biotechnology Faculty, Saratov State Agrarian University, 1 Teatralnaya Square, 410012 Saratov, Russia
[5] Moscow Institute of Physics and Technology, 9 Institutskiy per., 141701 Dolgoprudny, Russia
* Correspondence: nikelshparg.evelina.2010@post.bio.msu.ru (E.I.N.); bratashovdn@info.sgu.ru (D.N.B.)

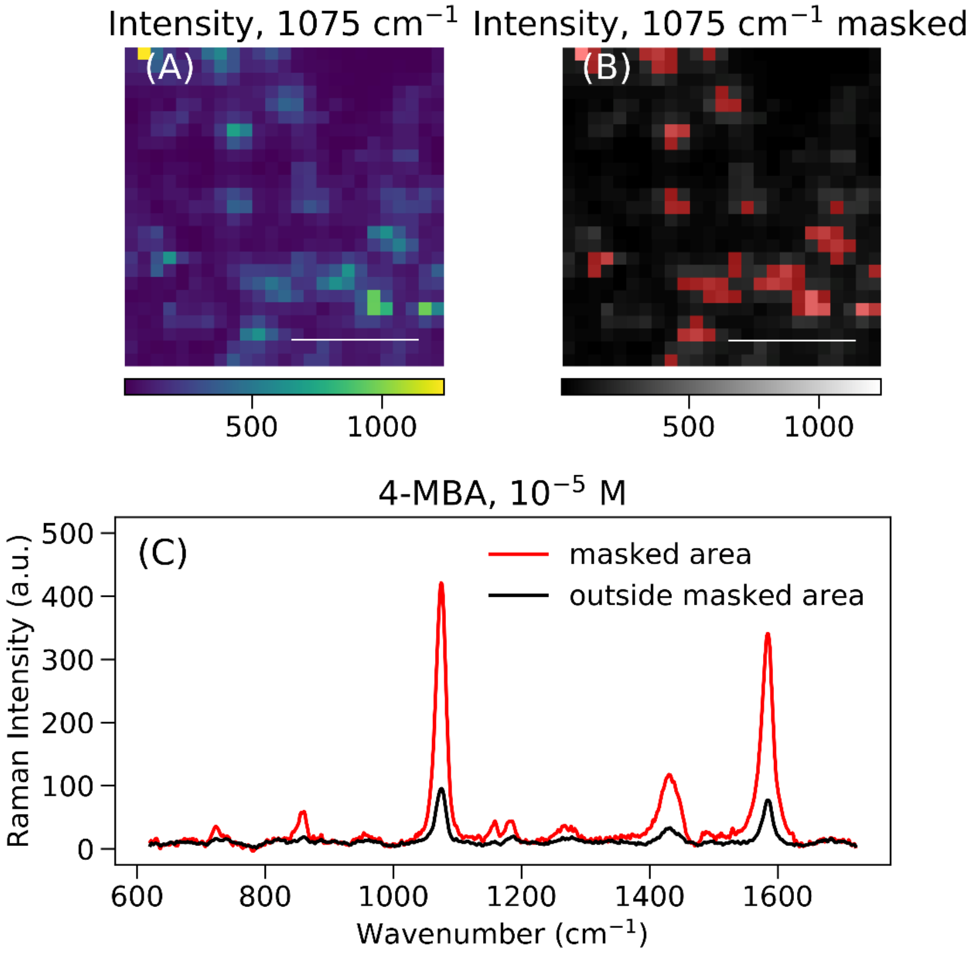

Figure S1. SERS map of 4-MBA (10^{-5} M solution in ethanol). (**A**) Intensity distribution at 1075 cm^{-1} peak. (**B**) The same map, values above threshold calculated using Otsu's method are marked with red. The scale bars at (**A**,**B**) correspond to 10 µm. (**C**) Mean spectra of area under (red line) and outside (black line) red mask (**B**).

Table S1. Raman peak assignment.

Wavenumber/cm^{-1}	Assignment	Reference
583–589	PO_4^{3-} in DNA and RNA	[1]
640–650	C–C twisting mode in proteins	[1–3]
645	[Fe–S]	[4]
660–670	Guanine, Thymine	[5,6]
680	Guanine	[7]
702	Cholesterol, cholesterol ester	[7]
720	Adenine, phosphatidylcholine, sphingomyelin	[8,9]
745	Thymine<br>H–C–O stretches in carbohydrates	[5,10]
750–755	Heme C (Fe^{3+})	[9]
760	Heme b (Fe^{3+})	[9]
780	Uracil and Cytosine ring breathing	[9]
789	Cytosine	[9]
802	Uracil	[6,9]
810–820	Proteins, DNA, RNA	[7]
832	RNA	[6]
900	Glycine	[3]
912	Carbohydrates	[5]
1000	Phenylalanine	[7]
1010	DPBS	[11]
1065–1067	Triacylglycerols, sphingomyelin;<br>DPBS	[8,9,11]
1080	Alkyl C–C stretches	[10]
1112–1115	C–C trans stretches in saturated lipids, polysaccharides	[10,12]
1120	Carbohydrates	[10]
1125	Triacylglycerols	[9]
1131	Triacylglycerols, sphingomyelin, phosphatidylcholine	[8,9]
1140–1170	ν(C–C) in lipids, proteins	[2,12]
1180	C–H bend in tyrosine, phenylalanine	[5]
1200	Proteins, DNA	[7]
1206	Heme b; Adenine, Thymine	[5,9]
1220–300	Amide III	[7,10]
1246	Amide III	[11]
1264	Adenine, Thymine	[5]
1270	=C–H cis stretch in unsaturated lipids, Amide III	[10]
1270	Unsaturated fatty acid	[8]
1300–1330 (broad)	Fatty acids	[8,9]
1310	Adenine	[6,9]
1326	Guanine	[6,9]
1330	Carbohydrates	[10]
1350	Proteins, lipids	[9]
1360	Tryptophan	[1]
1370	Adenine, Thymine, Guanine	[6]

1380–1390	unassigned	
1413	C=C stretching	[7]
1432	Adenine, Guanine	[6]
1445–1450	Alkyl C–H_2 bend mostly in lipids	[8,9]
1480	Adenine, Guanine	[6]
1490–1495	C–N stretching vibration	[7]
1510	Mostly cytosine	[5,9]
1535–50	Adenine, Guanine	[6]
1545	Heme B	[9]
1548	Heme C	[9]
1560	Tryptophan	[3]
1570	DNA, RNA, proteins	[5]
1595	Adenine	[6]
1600	C–C stretch	[5,12]

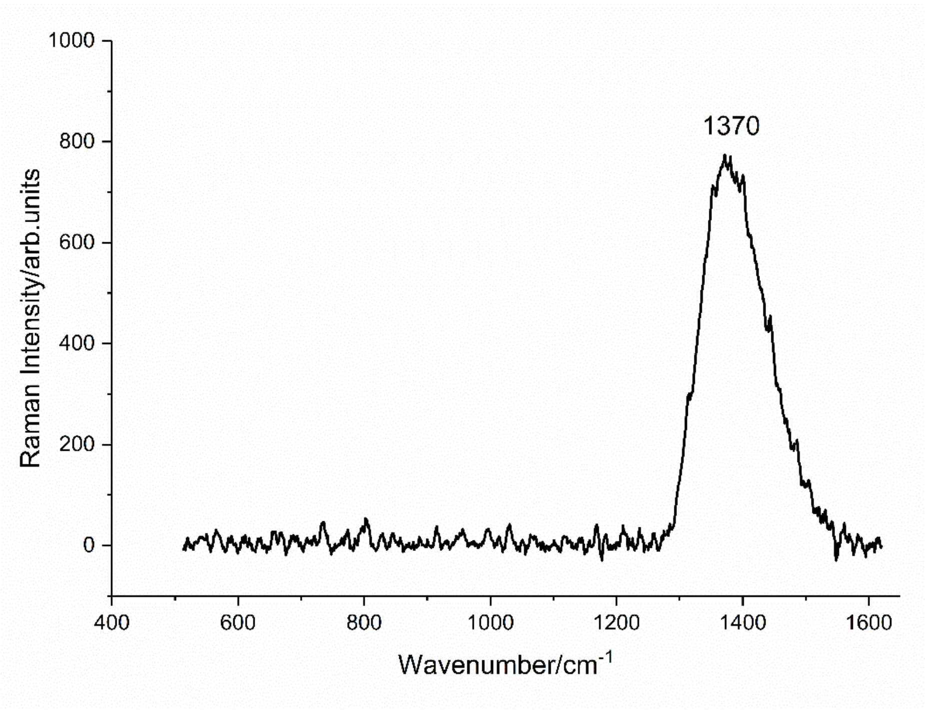

Figure S2. Spectrum of a cell outside gold substrates registered with 50×/0.5 n.a. objective lens, laser wavelength 785 nm, laser power 3 mW, acquisition time 1 s.

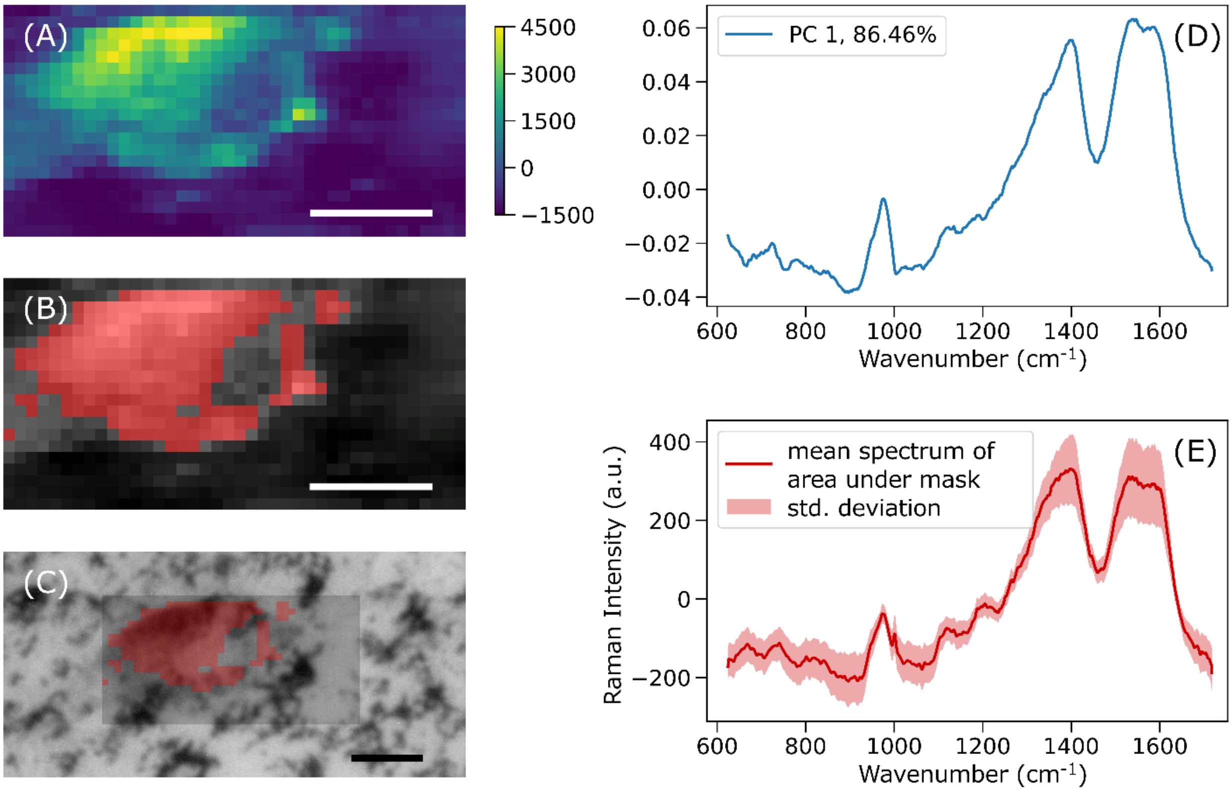

Figure S3. SERS mapping of B16-F10 cell. (**A**,**B**) 1st component in PCA decomposition (PC 1) score distribution. (**C**) White light image, scanning area is marked with light grey color. Red mask (**B**,**C**) corresponds to values (PC 1 score) above threshold calculated using Otsu's method. (**D**) PC 1 explaining 86.46% overall variance. (**E**) Mean spectrum of area under mask (**B**,**C**) with its standard deviation.

References

1. Cheng, W.-T.; Liu, M.-T.; Liu, H.-N.; Lin, S.-Y. Micro-Raman spectroscopy used to identify and grade human skin pilomatrixoma. *Microsc. Res. Tech.* **2005**, *68*, 75–79, doi:10.1002/jemt.20229.
2. Panikkanvalappil, S.R.; Hira, S.M.; Mahmoud, M.A.; El-Sayed, M.A. Unraveling the Biomolecular Snapshots of Mitosis in Healthy and Cancer Cells Using Plasmonically-Enhanced Raman Spectroscopy. *J. Am. Chem. Soc.* **2014**, *136*, 15961–15968, doi:10.1021/ja506289u.
3. Zhu, G.; Zhu, X.; Fan, Q.; Wan, X. Raman spectra of amino acids and their aqueous solutions. *Spectrochim. Acta Part A: Mol. Biomol. Spectrosc.* **2011**, *78*, 1187–1195, doi:10.1016/j.saa.2010.12.079.
4. Erjavec, N.; Pinato, G.; Ramser, K. Raman spectroscopy as a tool for detecting mitochondrial fitness. *J. Raman Spectrosc.* **2016**, *47*, 933–939, doi:10.1002/jrs.4930.
5. Chan, J.W.; Taylor, D.S.; Zwerdling, T.; Lane, S.M.; Ihara, K.; Huser, T. Micro-Raman Spectroscopy Detects Individual Neoplastic and Normal Hematopoietic Cells. *Biophys. J.* **2006**, *90*, 648–656, doi:10.1529/biophysj.105.066761.
6. Huefner, A.; Kuan, W.-L.; Barker, R.A.; Mahajan, S. Intracellular SERS Nanoprobes For Distinction Of Different Neuronal Cell Types. *Nano Lett.* **2013**, *13*, 2463–2470, doi:10.1021/nl400448n.
7. Movasaghi, Z.; Rehman, S.; Rehman, I.U. Raman Spectroscopy of Biological Tissues. *Appl. Spectrosc. Rev.* **2007**, *42*, 493–541, doi:10.1080/05704920701551530.
8. Krafft, C.; Neudert, L.; Simat, T.; Salzer, R. Near infrared Raman spectra of human brain lipids. *Spectrochim. Acta Part A: Mol. Biomol. Spectrosc.* **2005**, *61*, 1529–1535, doi:10.1016/j.saa.2004.11.017.
9. Brazhe, N.A.; Nikelshparg, E.I.; Prats, C.; Dela, F.; Sosnovtseva, O. Raman probing of lipids, proteins, and mitochondria in skeletal myocytes: a case study on obesity. *J. Raman Spectrosc.* **2017**, *48*, 1158–1165, doi:10.1002/jrs.5182.

10. Wu, H.; Volponi, J.V.; Oliver, A.E.; Parikh, A.; Simmons, B.; Singh, S. In vivo lipidomics using single-cell Raman spectroscopy. *Proc. Natl. Acad. Sci.* **2011**, *108*, 3809–3814, doi:10.1073/pnas.1009043108.
11. Fazio, B.; D'Andrea, C.; Foti, A.; Messina, E.; Irrera, A.; Donato, M.G.; Villari, V.; Micali, N.; Marago, O.M.; Gucciardi, P.G. SERS detection of Biomolecules at Physiological pH via aggregation of Gold Nanorods mediated by Optical Forces and Plasmonic Heating. *Sci. Rep.* **2016**, *6*, 26952, doi:10.1038/srep26952.
12. Sch Schulz, H.; Baranska, M. Identification and quantification of valuable plant substances by IR and Raman spectroscopy. *Vib. Spectrosc.* **2007**, *43*, 13–25, doi:10.1016/j.vibspec.2006.06.001.
